# Development of a high-sensitivity and short-duration fluorescence *in situ* hybridization method for viral mRNA detection in HEK 293T cells

**DOI:** 10.3389/fcimb.2022.960938

**Published:** 2022-10-04

**Authors:** Dailun Hu, Tao Wang, Jasim Uddin, Wayne K. Greene, Dakang Hu, Bin Ma

**Affiliations:** ^1^ Clinical College, Hebei Medical University, Shijiazhuang, China; ^2^ Telethon Kids Institute, Perth Children’s Hospital, Nedlands, WA, Australia; ^3^ Medical School, University of Western Australia, Nedlands, WA, Australia; ^4^ School of Veterinary Medicine, Murdoch University, Murdoch, WA, Australia; ^5^ Medical, Molecular and Forensic Sciences, Murdoch University, Murdoch, WA, Australia; ^6^ Department of Laboratory Medicine, Taizhou Municipal Hospital, Taizhou, China

**Keywords:** SARS-CoV-2, fluorescence *in situ* hybridization, mRNA, HEK 293T cell, RNA virus

## Abstract

Coronavirus disease 2019 (COVID-19) is an extremely contagious illness caused by severe acute respiratory syndrome coronavirus 2 (SARS-CoV-2). Early disease recognition of COVID-19 is crucial not only for prompt diagnosis and treatment of the patients, but also for effective public health surveillance and response. The reverse transcription-polymerase chain reaction (RT-PCR) is the most common method for the detection of SARS-CoV-2 viral mRNA and is regarded as the gold standard test for COVID-19. However, this test and those for antibodies (IgM and IgG) and antigens have certain limitations (e.g., by yielding false-negative and false-positive results). We have developed an RNA fluorescence *in situ* hybridization (FISH) method for high-sensitivity detection of SARS-CoV-2 mRNAs in HEK 293T cell cultures as a model. After transfection of HEK 293T cells with plasmids, Spike (S)/envelope (E) proteins and their mRNAs were clearly detected inside the cells. In addition, hybridization time could be reduced to 2 hours for faster detection when probe concentration was increased. Our approach might thus significantly improve the sensitivity and specificity of SARS-CoV-2 detection and be widely applied for the high-sensitivity single-molecular detection of other RNA viruses (e.g., Middle East respiratory syndrome coronavirus (MERS-CoV), Hepatitis A virus, all influenza viruses, and human immunodeficiency virus (HIV)) in various types of samples including tissue, body fluid, blood, and water. RNA FISH can also be utilized for the detection of DNA viruses (e.g., Monkeypox virus, human papillomavirus (HPV), and cytomegalovirus (CMV)) by detection of their mRNAs inside cells or body fluid.

## Introduction

Coronavirus disease 2019 (COVID-19) is an extremely contagious illness caused by severe acute respiratory syndrome coronavirus 2 (SARS-CoV-2; Borges do Nascimento et al., 2021; Aimrane et al., 2022; Al-Awwal et al., 2022). Recent evidence indicates over 430 million cases and 5.92 million deaths worldwide (Al-Awwal et al., 2022). The early disease recognition of COVID-19 is crucial not only for the prompt diagnosis and treatment of patients, but also for effective public health surveillance, containment, and response (Borges do Nascimento et al., 2021; Aimrane et al., 2022; Al-Awwal et al., 2022).

Coronaviruses, which include SARS-CoV-2, severe acute respiratory syndrome coronavirus 1 (SARS-CoV-1), and Middle East respiratory syndrome coronavirus (MERS-CoV), are a group of RNA viruses that can infect many different types of animals (including mammals and birds) and cause mild to severe respiratory infections (V’kovski et al., 2021; da Silva Torres et al., 2022). They are spherical enveloped viruses with a positive-sense single-stranded RNA genome (ranging from 26.4 to 31.7 kilobases) and a helically symmetrical nucleocapsid (N; V’kovski et al., 2021). At the 5′ end, the genomic RNA contains two large open reading frames (ORF; ORF1a and ORF1b) encoding 16 non-structural proteins. At the 3’ end, the genome encodes four structural proteins [spike (S), envelope (E), membrane (M), and N], and nine accessory proteins (ORF3a, 3b, 6, 7a, 7b, 8, 9a, 9b, and 10; V’kovski et al., 2021).

The polymerase chain reaction (PCR)-based method (including the reverse transcription-polymerase chain reaction (RT-PCR)) is the most commonly used for the detection of SARS-CoV-2 viral RNA in both symptomatic and asymptomatic patients and is considered the gold standard test for COVID-19 (Lin et al., 2015; Mardian et al., 2021; Rabaan et al., 2021; Yoo et al., 2021). The three main SARS-CoV-2-specific, highly conserved, and abundantly expressed genes targeted by RT-PCR are the ORF1ab, N, and E genes (Chu et al., 2020; Corman et al., 2020). However, RT-PCR has several limitations for SARS-CoV-2 detection. The first is the possibility that a false-negative result arises because of several factors ranging from sample collection to data interpretation (Mardian et al., 2021). False negatives have been reported in ~30% (range 10–40%) of patients with COVID-19 (Weissleder et al., 2020). Missed detection caused by false negatives therefore has severe consequences because a super-spreader might remain or be released into the community without further quarantine and/or treatment. Some patients only produce a positive result after a few negative results, significantly affecting and delaying follow-up treatments. The second limitation of RT-PCR is the possibility of a false-positive result attributable to technical errors (particularly contamination during sample collection and manual RT-PCR processing; Keaney et al., 2021; Mardian et al., 2021). The third is that the requirements for setting up an RT-PCR laboratory are usually high. An RT-PCR laboratory needs instruments capable of nucleic acid extraction and of carrying out quantitative fluorescence PCR; such instruments might only be available in some clinical laboratories (Hong et al., 2020). In addition, a level P2 laboratory as a minimum (plus P3 protection) is needed to avoid viral cross-contamination and infection of medical health professionals.

The COVID-19 Antibody (IgG and IgM) Test is a blood test that determines whether an individual has previously had a SARS-CoV-2 infection by the detection of antibodies against specific viral proteins (Lindsay et al., 2021; Ravi et al., 2021). As the first antibody to appear during infection, IgM is often utilized as a marker of acute infection. With the development of infection, the level of IgG increases, and the concentration of IgM gradually decreases, possibly disappearing after a certain time. Compared with the RT-PCR, the antibody test is more straightforward, faster, and more efficient (often showing strong sensitivity and specificity; Ravi et al., 2021). However, this test also has certain limitations. The first is the possibility of a false-negative result. For example, if the test is performed too early following an infection, a negative result may be obtained (Ravi et al., 2021). The second limitation is that specific IgM and IgG tests also suffer from false positives arising (Lindsay et al., 2021). For example, some weak positive results near the positive judgment value (cut-off value) are likely to be false positives. In addition, the presence of endogenous or exogenous interfering substances can lead to false positives. Furthermore, cross-reactivity is a significant challenge, since six other coronaviruses can also infect humans (Chia et al., 2020).

The COVID-19 Rapid Antigen Test (RAT) is an immunoassay for the qualitative detection of SARS-CoV-2 N antigen in nasal swabs and saliva (Wang et al., 2021; Gans et al., 2022; Khalid et al., 2022). These tests have moderate sensitivity and specificity for the detection of SARS-CoV-2. However, false-positive results are reported to be as high as 40% under certain conditions (Gans et al., 2022). Therefore, the sensitivity and specificity of the antigen assay are still inferior to those of the RT-PCR assay and might not match the requirements for clinical diagnosis and the screening of COVID-19 infections.

RNA fluorescence *in situ* hybridization (FISH; Ma et al., 2010; Ma and Tanese, 2013) has been applied to detect several RNA viruses including the influenza virus (Lakdawala et al., 2014). Since SARS-CoV-2 is a positive-sense single-stranded RNA coronavirus, our intention has been to develop a highly sensitive and reliable RNA-FISH method for the early accurate detection and screening of SARS-CoV-2 by using HEK 293T cell culture as a model system.

## Materials and methods

### Cell culture and transfection

Glass coverslips (round, 13mm diameter; ProSciTech, Kirwan, QLD, Australia) were briefly rinsed with 70% ethanol and then treated with 0.1 mg/L poly-D-lysine (PDL; Sigma, Bayswater, VIC, Australia) for 10 min. After being washed three times with distilled water, the coverslips were ready for the culture of HEK 293T cells (Sigma).

10,000-15,000 HEK293T cells [in 1.0 ml Dulbecco’s modified Eagle’s medium (DMEM; Sigma) with 10% fetal bovine serum (FBS; Thermo Fisher Scientific, Malaga, WA, Australia) and Penicillin (100 units/ml)-Streptomycin (100 µg/ml; Sigma)] were seeded on the PDL-coated glass coverslips in 24-well culture plates and cultivated in a humidified SANYO MCO-5AC incubator (SANYO, Osaka, Japan) at 37°C, supplemented with 5% CO_2_.

After reaching 70-80% confluency, cells were transfected by using the Lipofectamine™ 3000 Transfection Reagent (Thermo Fisher Scientific) according to the instructions of the manufacturer. In Tube 1, 0.75 µl Lipofectamine 3000 reagent was diluted in 25 µl Opti-MEM™ I Reduced Serum Medium (Thermo Fisher Scientific) for each well in a 24-well culture plate. In Tube 2, 200 ng pUNO1-SARS2-S (D614G) plasmid (*In vivo*Gen, San Diego, California, US), 200 ng pUNO1-SARS2-E plasmid (*In vivo*Gen), and 0.5 µl P3000™ reagent were added to 25 µl Opti-MEM™ I Reduced Serum Medium for each well. We used 400 ng plasmid for single transfection. The solution in Tube 2 solution was added to that in Tube 1. After being mixed well, the mixture was incubated for 10 min at room temperature and then added to the cell culture wells (50 µl/well). At various time points after transfection (e.g., 2 h, 4 h, 8 h, 16 h, and 24 h), cells were fixed and processed for further analysis.

### FISH

The steps for cell culture/transfection and FISH are shown in **Supplementary Figure 1**. Diethyl pyrocarbonate (DEPC)-treated water (ribonuclease-free water) was used for the preparation of phosphate-buffered saline (PBS) and other reagents (Ma and Tanese, 2013). Cells on glass coverslips were rinsed briefly in PBS, fixed with 4% paraformaldehyde (PFA; Electron Microscopy Sciences, Hatfield, PA, USA) for 10 min, and then washed three times with PBS. The cells were subsequently permeabilized with 0.25% Triton X-100 (Sigma) in PBS for 5 min.

After a 5-min rinse with 1× sodium chloride/sodium citrate (1 × SSC), coverslips (upside down on a paraffin film in a humidified box) were incubated in 40 μl hybridization buffer [25% dextran sulfate (Sigma), 40% formamide (Sigma), 30 μg/ml single-stranded salmon sperm DNA (Sigma), 30 μg/ml yeast tRNA (Sigma), 0.4% bovine serum albumin (BSA; Sigma), 20 mM ribonucleoside vanadyl complex (Sigma), 0.01 M sodium phosphate buffer (pH 7.0), 2 × SSC] in an Extron HI 2001 hybridization oven (Bartelt Instruments, Heidelberg West, Victoria, Australia) for 1 h at 37°C for pre-hybridization. The cells were then hybridized with probes (single probe: 400 ng; mixed probes: 200 ng Probe 1 + 200 ng Probe 2) diluted in 40 μl hybridization buffer in the hybridization oven for 4 h at 37°C. The sequences and sources of digoxin (DIG)-labeled probes are shown in **Table 1**. For experiments with a 2-h hybridization time, we utilized 800 ng probe (400 ng Probe 1 + 400 ng Probe 2) in 40 μl hybridization buffer. As a positive control for our FISH method, an Oligo dT probe (targeting the poly-A tail of all mRNAs) was used to detect the total mRNAs in the HEK 293T cells.

**Table 1:** Sequences and sources of probes used for FISH.

Probe	Sequences	Company
BME-001	CACTAGCCATCCTTACTGCGCTTCGATTGTGTGCGTACTGCTGC/3DiG_N/	Integrated DNA Technologies(Coralville, Iowa, United States)
BME-002	GCCATCCTTACTGCGCTTCGATTGTGTGCGTACTGCTGCA/3DiG_N/	Integrated DNA Technologies
BMS-001	TGGCCATGGTACATTTGGCTAGGTTTTATAGCTGGCTTGATTGCCATAGT/3DiG_N/	Integrated DNA Technologies
BMS-002	GCACACGCCTATTAATTTAGTGCGTGATCTCCCTCAGGGT/3DiG_N/	Integrated DNA Technologies
Oligo dT	Single-stranded sequence of deoxythymine (dT), 24mer-DIG	Life Technologies, Grand Island, NY

After hybridization, cells were washed with 40% formamide/1 × SSC for 30 min at 37°C with gentle shaking (in the hybridization oven), followed by washes for 3 × 10 min in 1 × SSC with gentle shaking on an orbital shaker at room temperature.

### Antibodies

The specificities and sources of antibodies are described in **Table 2**.

**Table 2:** Specificities and sources of primary and secondary antibodies.

Target	Conjugate	Species and isotype	Company
Digoxin(CDIG-65A)		Chick IgY, polyclonal	Immunology Consultants Laboratory (Portland, OR, USA)
SARS-CoV-2 1/2 Spike protein(2B3E5)		Mouse IgG, monoclonal	Cell Signaling Technology (Danvers, MA, United States)
SARS-CoV-2 E protein		Rabbit IgG, polyclonal	Abcam Australia (Melbourne, Australia)
Chick IgG	Alexa Fluor^®^ 488	Goat polyclonal	Abcam Australia
Chick IgG	Alexa Fluor^®^ 555	Goat polyclonal	Abcam Australia
Rabbit IgG H&L	Alexa Fluor^®^ 488	Goat polyclonal	Abcam Australia
Rabbit IgG H&L	Alexa Fluor^®^ 555	Goat polyclonal	Abcam Australia
Mouse IgG	Alexa Fluor^®^ 647	Goat polyclonal	Abcam Australia

### Detection of DIG-labeled probes and immunofluorescence staining

Coverslips with cells were rinsed briefly with PBS. All washes (3×5 min) between steps were performed with PBS at room temperature. Cells were then incubated with antibody dilution buffer [2% goat serum (Sigma) in PBS] for 20 min at room temperature to block any potential non-specific binding sites to the antibodies. The cells of the experimental group were incubated with primary antibodies for 1 h at room temperature. Primary antibodies were omitted in negative controls.

Cells were then incubated with secondary antibodies for 45 min at room temperature. Finally, coverslips (upside down) were mounted on microscope slides (25mm× 75mm; ProSciTech) by means of Fluorescence Mounting Medium (DAKO, North Sydney, Australia).

### Confocal microscopy and image processing

Confocal microscopy was carried out on a Nikon C2 Plus Confocal Microscope (Nikon Instruments, Melville, NY, USA) with three lasers (488 nm, 561 nm, and 633 nm). A Plan Apo λ 60x/1.40 oil immersion objective lens was utilized for all imaging. The operation program for the confocal microscope was NIS-Elements AR. Maximal intensity projection of a Z-stack was performed by using the “Maximal intensity projection” of the NIS-Elements AR program. 3D reconstruction was performed by using the “Volume rendering” in the NIS-Elements AR program. The images were then saved as bitmap (BMP) image files and further edited (cropping and labeling) by using Corel PaintShop Pro 2020 (Corel, Ottawa, Canada).

## Results

### Co-detection of SARS-CoV-2 E/S and their mRNAs

**Figure 1:**
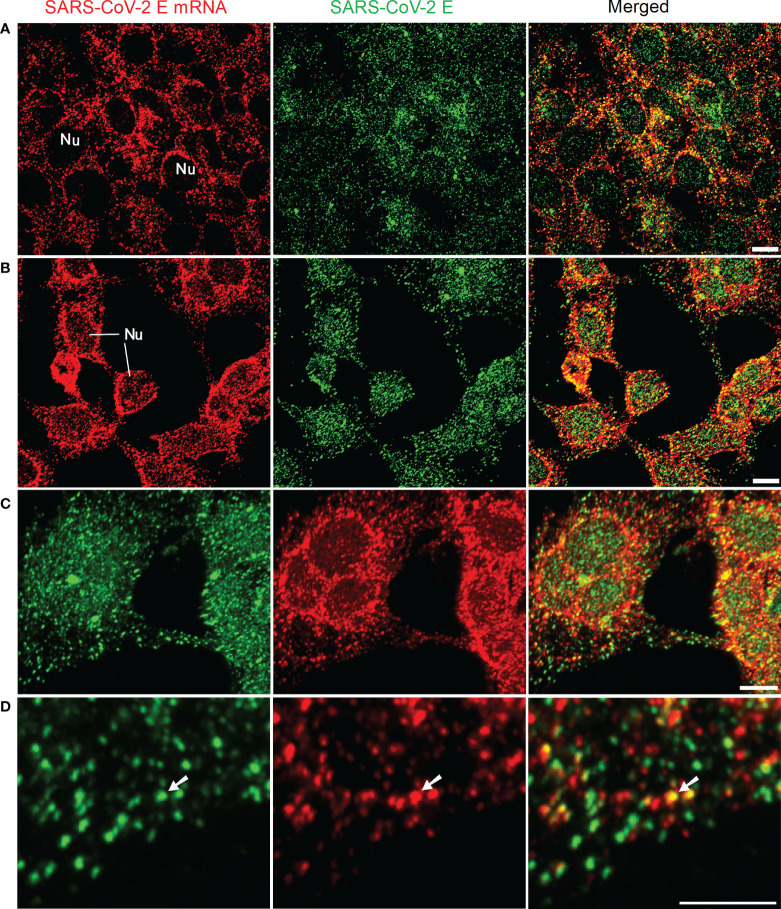
Detection of SARS-CoV-2 E mRNA (with single probe-BME001; red) and SARS-CoV-2 E protein (green) in HEK 293T cells at 24 h after transfection with SARS-CoV-2 E plasmids. The yellow color in merged images shows the colocalization of SARS-CoV-2 E and its mRNA **(A)** 2D images for E mRNA and E protein. **(B)** Images are maximal intensity projections of a Z-Stack. Optical slice interval: 0.50 µm; Stack size: 6.0 µm. **(C)** 3D projection by using the Z-stack in (B; cropped). **(D)** High-resolution view of images in (C; cropped). The white arrows indicate the colocalization of E mRNA and E protein inside the cells. Objective lens: 60×; Scale bar: 10 µm; Nu, nucleus.

At first, we tested our mRNA FISH by using a single DNA probe for SARS-CoV-2 E mRNA. HEK 293T cells were transfected with SARS-CoV-2 E plasmids. At 24 h after transfection, SARS-CoV-2 E mRNA and protein were detected using FISH and immunofluorescent staining, respectively. The results are shown in **Figure 1**. Abundant E mRNAs (demonstrated by solid granular staining) were observed inside the HEK 293T cells. Furthermore, colocalization of E protein and mRNA (appearing yellow in the merged image) indicated the E protein being translated (**Figure 1**). We also utilized optic sectioning and 3D reconstruction for a better demonstration of E mRNA and protein inside the HEK 293T cells (**Figure 1B-D** and **Supplementary Video 1**).

**Figure 2:**
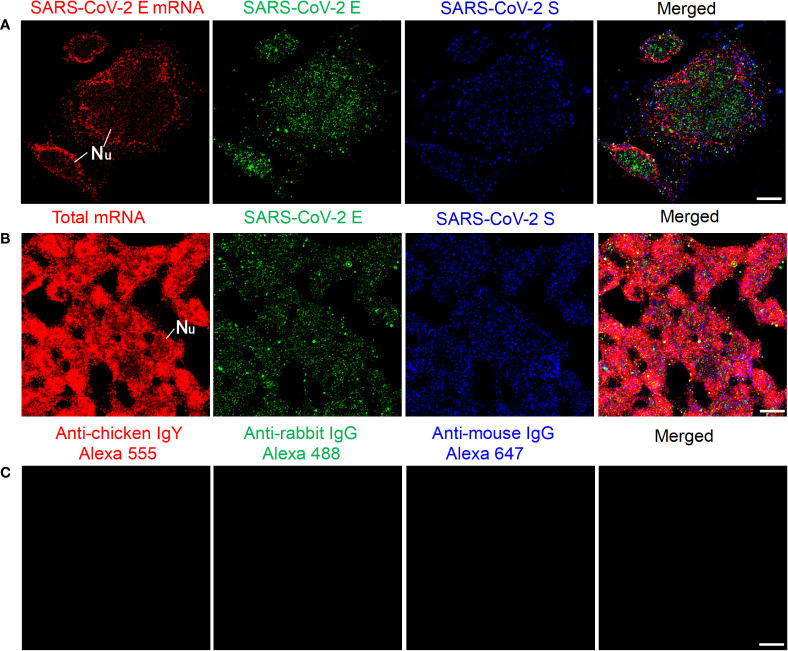
Detection of SARS-CoV-2 E mRNA and E/S protein in HEK 293T cells at 24 h after transfection with SARS-CoV-2 E and S plasmids. **(A)** SARS-CoV-2 E mRNA (with mixed probes containing BME001 and BME002), SARS-CoV-2 E, and SARS-CoV-2 S are shown in red, green, and blue, respectively. **(B)** Total mRNAs (detected by Oligo dT probes), SARS-CoV-2 E, and SARS-CoV-2 S are shown in red, green, and blue, respectively. **(C)** Negative controls by using secondary antibodies. Objective lens: 60×; Scale bar: 10 µm; Nu, nucleus.

We then tested our mRNA FISH by using two DNA probes for SARS-CoV-2 E mRNA. HEK 293T cells were transfected with SARS-CoV-2 E and S plasmids, and 24 h after transfection, SARS-CoV-2 E mRNAs and E/S proteins were detected by using FISH and immunofluorescent staining, respectively. The results are shown in **Figure 2A**. We also observed abundant SARS-CoV-2 E mRNA (solid granular staining) together with SARS-CoV-2 E/S protein (solid granular staining) inside the cells. As a positive control for our FISH method, an Oligo dT probe (targeting the poly-A tail of all mRNAs) was used to detect the total mRNAs in the HEK 293T cells, and the results are shown in **Figure 2B**. In negative controls, no fluorescent signals were observed when only secondary antibodies were applied (**Figure 2C**).

**Figure 3:**
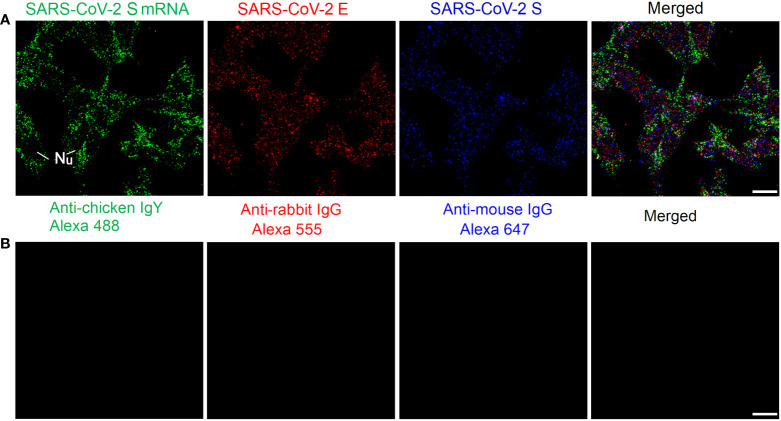
Detection of SARS-CoV-2 S mRNA and S/E proteins in HEK 293T cells at 24 h after transfection with SARS-CoV-2 S and E plasmids. **(A)** SARS-CoV-2 S mRNA (with mixed probes containing BMS001 and BMS002), SARS-CoV-2 E, and SARS-CoV-2 S are shown in green, red, and blue, respectively. **(B)** Negative controls by using secondary antibodies. Objective lens: 60×; Scale bar: 10 µm; Nu, nucleus.

We also tested our mRNA FISH approach by using two DNA probes for SARS-CoV-2 S mRNA. HEK 293T cells were transfected with SARS-CoV-2 S and E plasmids. At 24 h after transfection, SARS-CoV-2 S mRNAs and S/E proteins were detected by using FISH and immunofluorescent staining, respectively. The results are shown in **Figure 3A**. We observed abundant SARS-CoV-2 S mRNA (solid granular staining) together with SARS-CoV-2 E/S protein (solid granular staining) inside the cells. In negative controls for secondary antibodies, no fluorescent signals were observed when primary antibodies were omitted (**Figure 3B**).

### FISH of SARS-CoV-2 S mRNAs at various time points after transfection

**Figure 4:**
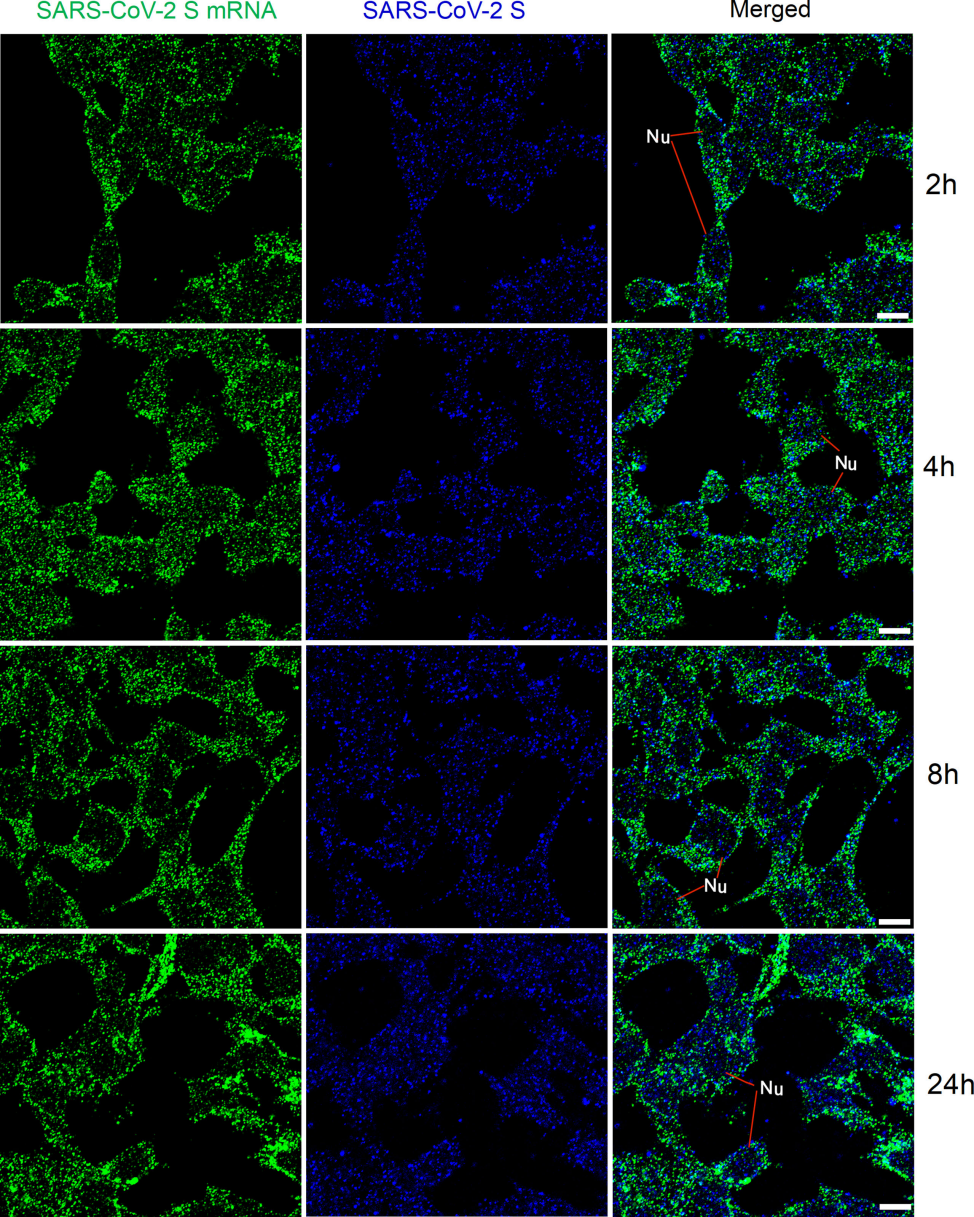
Detection of SARS-CoV-2 S mRNA (with mixed probes containing BMS001 and BMS002; green) and SARS-CoV-2 S (blue) in HEK 293T cells after transfection (2 h, 4 h, 8 h, and 24 h) with SARS-CoV-2 S plasmids. Objective lens: 60×; Scale bar: 10 µm; Nu, nucleus.

We then checked whether, after transfection, mRNAs could be detected at various time points mimicking viral replication in the human body. HEK 293T cells were transfected with SARS-CoV-2 S plasmids. At 2 h, 4 h, 8 h, and 24 h after transfection, SARS-CoV-2 S mRNA and protein were detected using FISH and immunofluorescent staining, respectively. The results are shown in **Figure 4**. SARS-CoV-2 S mRNA was clearly demonstrated by the solid granular staining inside HEK 293T cells at all time points. In addition, the S protein demonstrated by granular staining was also observed inside HEK 293T cells at all time points. Therefore, SARS-CoV-2 mRNA and protein could be observed as early as 2 h after transfection with S plasmid.

### FISH of SARS-CoV-2 mRNAs with reduced hybridization time

**Figure 5:**
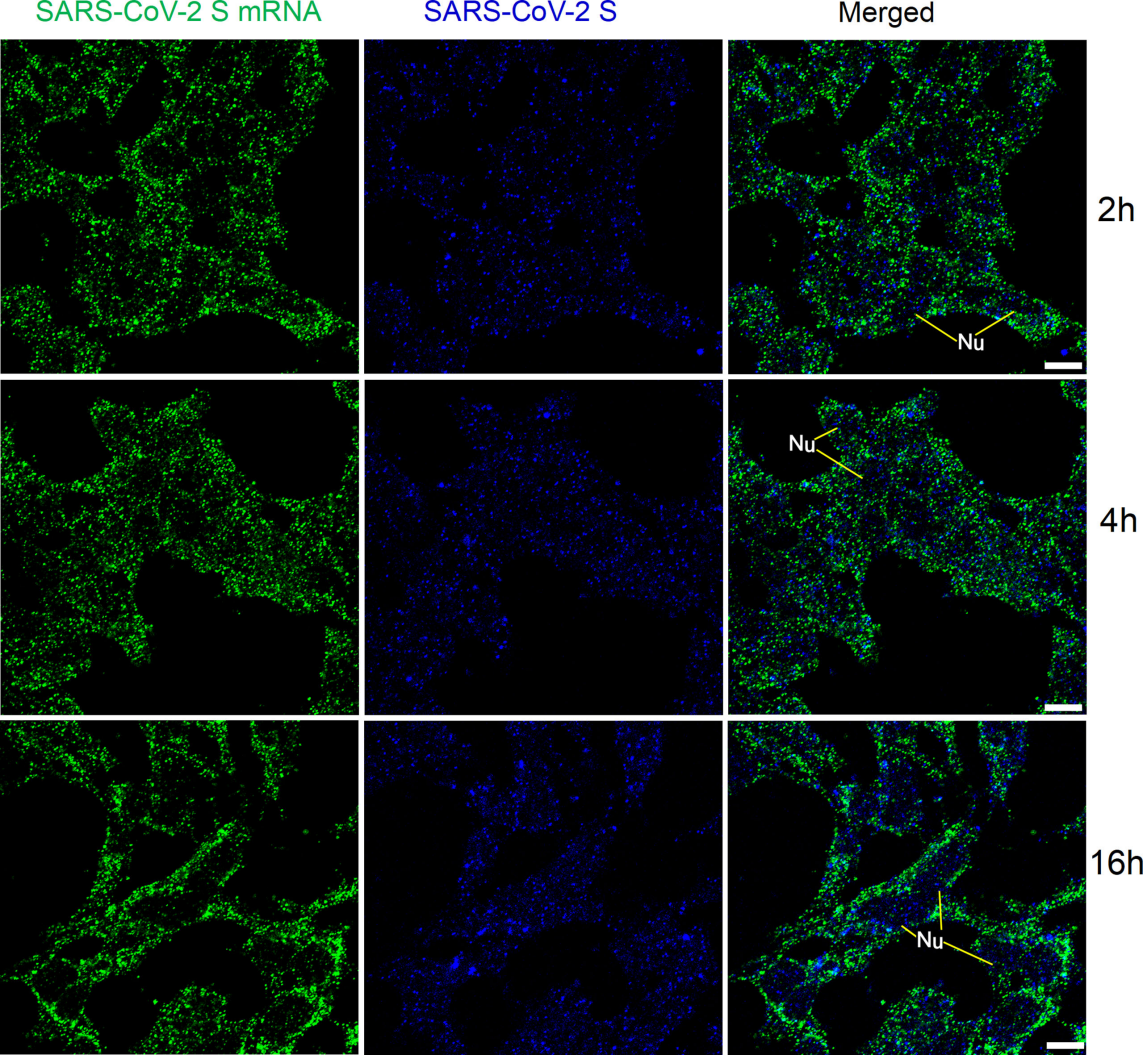
Detection of SARS-CoV-2 S mRNA (with mixed probes containing BMS001 and BMS002; with 2 h hybridization time; green) and SARS-CoV-2 S (blue) in HEK 293T cells after transfection (2 h, 4 h, and 16 h) with SARS-CoV-2 S plasmids. Objective lens: 60×; Scale bar: 10 µm; Nu, nucleus.

We then checked whether the time for pre-hybridization and hybridization could be reduced to achieve faster detection of viral mRNAs. At 2 h, 4 h, and 16 h after transfection with SARS-CoV-2 S plasmid, HEK 293T cells were pre-hybridized for 30 min and then hybridized with an increased amount of probes (800 ng in total) for 2 h. The results are shown in **Figure 5**. We observed solid granular FISH signals for SARS-CoV-2 S at all time points, together with granular staining signals for SARS-CoV-2 S protein.

**Figure 6:**
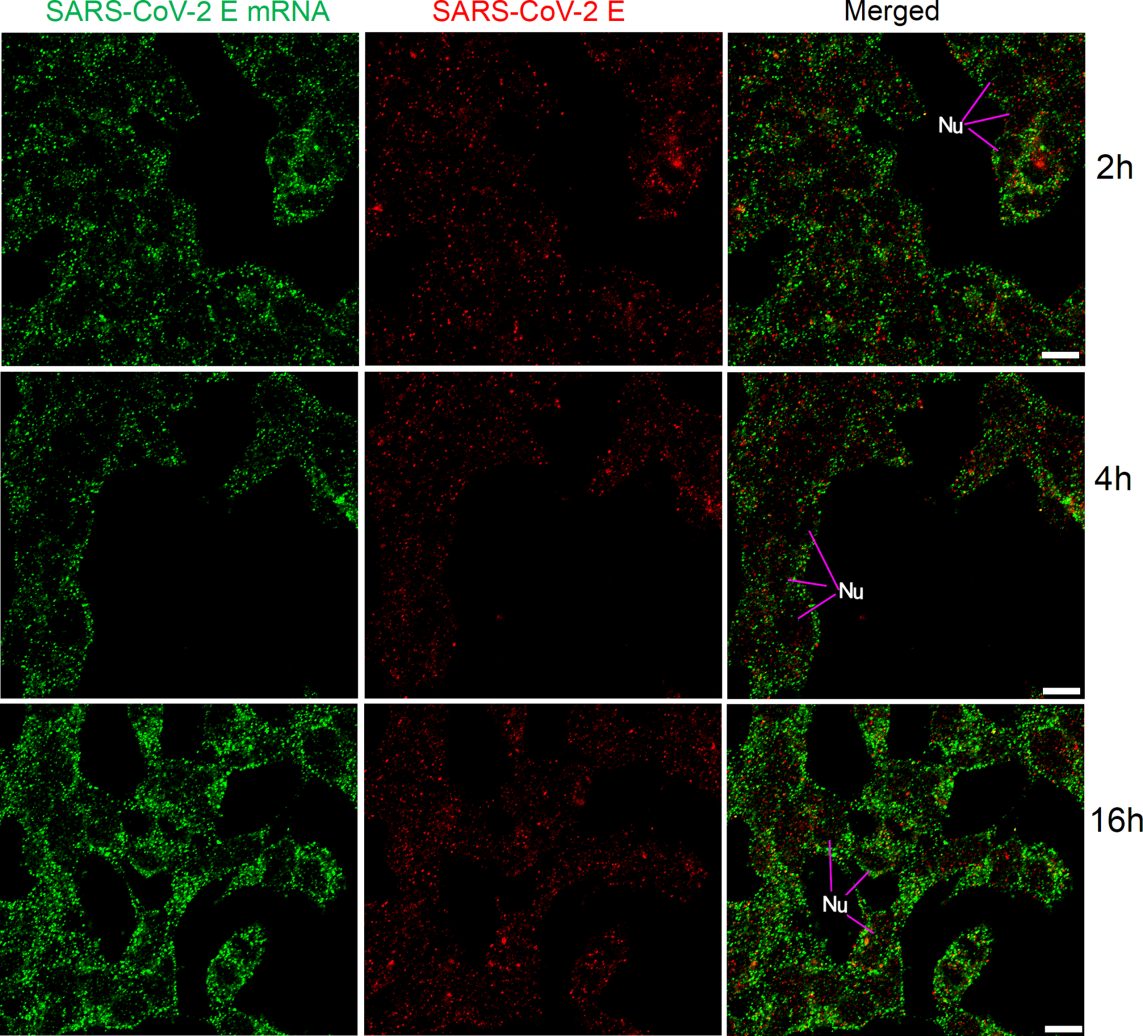
Detection of SARS-CoV-2 E mRNA (with mixed probes containing BME001 and BME002; with 2 h hybridization time; green) and SARS-CoV-2 E protein (red) in HEK 293T cells after transfection (2 h, 4 h, and 16 h) with SARS-CoV-2 E plasmids. Objective lens: 60×; Scale bar: 10 µm; Nu, nucleus.

We also applied the same FISH protocol (with reduced pre-hybridization and hybridization times) for SARS-CoV-2 E mRNA, and the results are shown in **Figure 6**. SARS-CoV-2 E mRNA was detectable inside HEK 293T cells at all time points (similar to the SARS-CoV-2 S mRNA) after transfection. Although S mRNA and E mRNA were readily detectable with reduced pre-hybridization and hybridization times, the resulting images (shown in **Figures 5**-**6**) were not as sharp as those shown in **Figures 1**-**3**.

## Discussion

We have developed an RNA FISH approach as a high-sensitivity single-molecular (particle) detection method for viral mRNAs (including S and E) inside HEK 293T cells. Short DNA probes (about 40-50 nt) have been utilized for the detection of mRNAs, and this method enables us to conduct qualitative, quantitative, and cellular localization/analysis of RNAs by using fluorescent or confocal microscopy. Although our method can detect single-molecule mRNA (or mRNA fragments) inside the cells, the observed granular staining might contain a few mRNAs, since SARS-CoV-2 viral RNA replicates within double-membrane vesicles (DMVs; Gómez et al., 2021) in the cytoplasm of HEK 293T cells. These DMVs are similar to the mRNA granules that we have described in previous studies, since both of them might contain a few mRNA molecules (Ma et al., 2010; Ma et al., 2012; Lakdawala et al., 2014; Gómez et al., 2021). We have established this method by using cell culture on coverslips, similar to the smears made from nasopharyngeal or oropharyngeal swabs.

### RNA FISH might improve sensitivity and reduce false negatives

In our previous studies, we have compared RT-PCR and mRNA FISH in the detection of mRNA/RNAs (Ma et al., 2012). PCR is an extremely sensitive detection method since it can amplify a specific region of DNA sequence 10^6^ times *in vitro*. To detect fluorescent signals, a threshold must be set to distinguish signal from noise (Mardian et al., 2021; Rabaan et al., 2021). Although RT-PCR is extremely sensitive, The Centers for Disease Control and Prevention (CDC) assays present detection limits ranging from 85 to 499 copies/ml, depending on the extraction method and the thermocycler used (Fung et al., 2020).

In RNA FISH, there are no requirements for RNA extraction, reverse transcription, or DNA amplification. FISH enhances the hybridization signal and improves the sensitivity through multiple immunochemical reactions, and its sensitivity is comparable with that of radioactive probes (Ma et al., 2012). Thus, RNA FISH enables high-sensitivity single-molecular (particle) detection of virus/viral RNA/RNA granules. The sensitivity of viral detection is therefore improved leading to a more accurate diagnosis.

In RT-PCR, the cycle threshold (Ct) value is the cycle number at which the fluorescence generated within a reaction crosses the fluorescence threshold, i.e., the fluorescent signal is significantly above that of the background fluorescence. It is inversely proportional to the original relative expression level of the gene of interest. However, Ct values might be affected by pre-analytic, analytic, and post-analytic variables (such as collection technique, specimen type, sampling time, viral kinetics, transport/storage conditions, nucleic acid extraction, viral RNA load, primer designing, real-time PCR efficiency, thermocycler used, and Ct value determination method; Mardian et al., 2021; Rabaan et al., 2021). In addition, Ct threshold values can demonstrate wide variation across differing populations and over time (Walker et al., 2021). For data interpretation, a Ct value smaller than 40 for all target genes is normally defined as a positive test (Mardian et al., 2021). Therefore, extra care should be taken when interpreting the results, especially when the Ct value is near the cut-off value. In RNA FISH, no cut-off value similar to the Ct is needed to distinguish between positive and negative results. Nevertheless, a threshold for differentiating fluorescent signal from noise (similar to RT-PCR) is needed. This type of threshold can be set up by the use of appropriate negative controls in the FISH. In our study, we performed these controls and obtained a clear background (low noise level).

RAT typically targets the SARS-CoV-2 N gene, which is not a mutation hotspot. A false-negative result might occur if the N antigen level is below the detection limit of the test. Moreover, RAT has been reported only to have high sensitivity when the viral load is high (e.g., 90% when 20 ≤ Ct ≤25). For example, detection of the viral antigen might be difficult during early infection or during the incubation period. When Ct is larger than 25, the sensitivity of RAT might only be 10% (Barrera-Avalos et al., 2022). In addition, RAT sensitivity might be much lower in asymptomatic or child patients (Brümmer et al., 2021; Fujita-Rohwerder et al., 2022). Therefore, RAT might have some limitations in the clinical diagnosis of SARS-CoV-2, although it is much faster and more convenient than RT-PCR or RNA FISH. Our RNA FISH might overcome these problems and detect viral mRNA at each period/stage of infection/disease.

### RNA FISH might improve specificity and reduce false positives

SARS-CoV-2 RT-PCR testing is associated with a small number of false-positive results that are normally caused by cross-contamination or/and non-specificity of primers (Keaney et al., 2021; Layfield et al., 2021; Mardian et al., 2021). Since RT-PCR can amplify a DNA sequence 10^6^ times, even a few virus/viral mRNAs can cause false-positive results. Cross-contamination might occur during sample collection (e.g., large-scale nucleic acid test) or sample preparation (e.g., negative samples are contaminated by strongly positive samples nearby or a healthy person inhales a few viruses floating in the air at the testing site; Layfield et al., 2021). In RNA FISH, viral RNA/genome from cross-contamination cannot be amplified, since there is no nucleic acid amplification step. In addition, smears made from nasopharyngeal or oropharyngeal swabs can be used for RNA FISH, and it is improbable that the virus/viral mRNAs from contamination will appear inside the cells.

The non-specificity of primers in RT-PCR is usually attributable to the quality of the manufacturer’s reagent (Westhaus et al., 2021). This problem might also be present in RNA FISH. However, the lengths of the probes that we have used in RNA FISH are about 40-50 nt, which is longer than those of regular RT-PCR primers (about 20 nt). Longer probes will have better specificity than shorter probes, although the hybridization/reaction time might be longer.

False positives might also be a problem in RAT in some circumstances. RAT can be more reliably applied in areas with community prevalence (e.g., the positive rate is higher than or equal to 5%). In low-endemic or non-endemic areas, false-positive results (up to 60%) are more likely to occur if RT-PCR is used as a “gold standard” (Gans et al., 2022).

### RNA FISH allows subcellular localization and analysis

PFA fixation of infectious samples can improve both biosafety and the speed of detection, while preserving the ultrastructure of biological material without interfering significantly with the preparation (i.e., negative staining) and the detection of viruses. Fixed samples can be kept for a long time (e.g., for retrospective analysis), and the infection risk of medical professionals is minimalized.

Subcellular localization and the analysis of RNA/mRNA in cells/tissues are not possible with RT-PCR. Our RNA FISH method can detect the uncoated RNA genome, replicating mRNA/RNA (by detecting the negative-strand mRNAs), and RNAs in viral particles inside cells and tissue fluid (e.g., sputum and saliva). Therefore, RNA FISH enables the detection of viral replication and of active infection. In addition, the cellular localization of mRNA/RNA can exclude some false positives since virus/viral RNA from cross-contamination is unlikely to be localized inside the cells.

The Ct values of RT-PCR can be correlated with viral load and disease severity in COVID-19 (Rabaan et al., 2021). However, no quantitative analysis can be performed by RT-PCR for RNA inside the samples. Our RNA FISH can localize mRNA/RNA inside the cells on smears so that both qualitative and quantitative analyses are possible (Ma et al., 2012). Quantitative analysis can be performed by examining the fluorescent intensity or the number of granules/DMVs containing RNA inside the cells on the smears (Rabaan et al., 2021). In addition, for smears without cells (e.g., smears prepared from saliva or wastewater), absolute quantitative analysis can be performed in order to obtain the number or concentration of viruses.

The determination of RT-PCR results is automatic, whereas result determination in RNA FISH is manually performed by pathologists. Nevertheless, we can also use artificial intelligence technology and digital pathology for automatic FISH and image analysis/result determination (van der Logt et al., 2015).

### FISH might be cost-effective and convenient

The cost of RNA FISH is much lower than that of RT-PCR. RNA FISH does not require a large amount of primers and enzymes for amplification, and so the cost might be lower than that of RT-PCR. In addition, RNA FISH does not need expensive instruments such as automated DNA/RNA purification systems, thermocyclers, and RT-PCR Detection Systems. A wide-field fluorescent microscope is sufficient for the final examination of slides enabling laboratory tests to be carried out in a regular laboratory with no sophisticated facilities and instruments [e.g., in small clinics/hospitals, in rural/remote/undeveloped regions (i.e., Australian Quarantine Centre at Christmas Island), or in some military bases].

The time required for RNA FISH is a little longer than that for RT-PCR because of the multiple steps involved including fixation, FISH, and immunodetection. However, we can use a few approaches to reduce the time needed. For example, in this study, we have reduced the hybridization time to 2 h and have still obtained excellent results. In addition, we can use directly fluorescent dye-labeled probes for direct hybridization, possibly reducing the time further. Furthermore, both RNA FISH and result determination can be automatized to reduce test time and labor (van der Logt et al., 2015).

Another limitation of mRNA FISH might be unspecific staining or background attributable to either non-specific antibody binding to endogenous Fc receptors (FcRs) or a combination of ionic and hydrophobic interactions (Buchwalow et al., 2011). If a primary antibody (e.g., anti-DIG) binds epitopes other than its target, it can generate unspecific signals that can be further amplified by dye-conjugated secondary antibodies. However, several approaches [e.g., use of highly specific antibodies, optimization of the antibody dilutions, use of negative controls for secondary antibodies, employment of F(ab’)_2_ fragments of antibodies only, and use of high-quality goat serum or BSA for blocking before immunostaining] can be utilized to minimize this effect (Ma and Tanese, 2013).

In summary, our approach might significantly improve the accuracy and sensitivity of SARS-CoV-2 detection. In the present work, we have only compared our method with other methods from methodological viewpoints. We have applied our mRNA FISH on several clinical samples (smears made from nasopharyngeal or oropharyngeal swabs) successfully. However, we have not tested its sensitivity and specificity for clinical diagnosis, because of biosafety/ethical issues.

Our RNA FISH can be performed on smears containing cells (e.g., from nasopharyngeal swabs) or smears without cells (e.g., from sputum, saliva, and wastewater). It can also be widely used for the high-sensitivity single-molecular detection of other RNA viruses [e.g., SARS-CoV-1, MERS-CoV, Hepatitis A virus, all influenza viruses, and human immunodeficiency virus (HIV)] in various type of samples (including tissue, body fluid, blood, and water). In addition, our RNA FISH can also be utilized for the detection of DNA viruses [e.g., Monkeypox virus, human papillomavirus (HPV), and cytomegalovirus (CMV)] *via* the detection of their mRNAs inside cells. We believe that our RNA FISH approach will increasingly find applications for more accurate diagnosis and more effective public health surveillance of viral infectious diseases.

## Data availability statement

The original contributions presented in the study are included in the article/**Supplementary Material**. Further inquiries can be directed to the corresponding authors.

## Author contributions

WG, DakH, and BM conceived the study and designed the experiments. DakH and BM wrote the paper. DaiH, TW, JU, and BM performed experiments and analyzed the data. All authors contributed to the article and approved the submitted version.

## Conflict of interest

The authors declare that the research was conducted in the absence of any commercial or financial relationships that could be construed as a potential conflict of interest.

## Publisher’s note

All claims expressed in this article are solely those of the authors and do not necessarily represent those of their affiliated organizations, or those of the publisher, the editors and the reviewers. Any product that may be evaluated in this article, or claim that may be made by its manufacturer, is not guaranteed or endorsed by the publisher.

## Supplementary material

Supplementary Figure 1 Flowchart of experimental design. Washing steps are not shown. PFA; paraformaldehyde; DIG; digoxin.

Supplementary Video 1 Detection of SARS-CoV-2 E mRNA (with probe-BME001; red) and SARS-CoV-2 E (green) in HEK 293T cells at 24 h after transfection with SARS-CoV-2 E plasmids. The video was generated by volume rendering of a Z-Stack (as shown in **Figure 1B**; cropped). Optical slice interval: 0.50 µm; Stack size: 6.0 µm. Objective lens: 60×.
