# Supplementary material for: Development of a high-sensitivity and short-duration fluorescence in situ hybridization method for viral mRNA detection in HEK 293T cells
Source: Front Cell Infect Microbiol. 2022 Oct 4;12:960938. doi: 10.3389/fcimb.2022.960938 (PMC9577401; doi:10.3389/fcimb.2022.960938)
Supplement: Supplementary Figure 1 — Flowchart of experimental design. Washing steps are not shown. PFA; paraformaldehyde; DIG; digoxin. [file Table_1.docx]

| HEK 293T cell culture  on glass coverslips |
| --- |
| Transfection with SARS-CoV-2  S/E plasmids (24 h or at different times) |
| Fixation with PFA |
| Permeabilization with Triton X-100 |
| Prehybridization |
| Hybridization with DIG-labeled probes  (4 h or reduced to 2 h) |
| Incubation with anti-DIG and  anti-S/E primary antibodies |
| Incubation with fluorescent dye-conjugated  secondary antibodies |
| Mounting |
| Confocal microscopy |
| Image analysis and 3D reconstruction |
